# Coping with ‘the grey area’ of antibiotic prescribing: a theory-informed qualitative study exploring family physician perspectives on antibiotic prescribing

**DOI:** 10.1186/s12875-022-01806-8

**Published:** 2022-07-28

**Authors:** Michelle Simeoni, Marianne Saragosa, Celia Laur, Laura Desveaux, Kevin Schwartz, Noah Ivers

**Affiliations:** 1 Public Health Ontario, Toronto, ON Canada; 2 Mount Sinai, Toronto, ON Canada; 3 Women’s College Hospital Institute for Health System Solutions and Virtual Care, Women’s College Hospital, Toronto, ON Canada; 4 Institute for Better Health, Trillium Health Partners, Toronto, ON Canada; 5 Dalla Lana School of Public Health, University of Toronto, Toronto, ON Canada

**Keywords:** Antibiotic Prescribing, Antimicrobial Resistance, Family Physician, Primary Care, Theoretical Domains Framework, Upper Respiratory Tract Infection

## Abstract

**Background:**

Unnecessary antibiotic use is associated with adverse side effects and rising rates of resistance at the individual and population level. This study used a theory-informed approach to identify potentially modifiable determinants of antibiotic prescribing for patients presenting to primary care with upper respiratory tract infection symptoms.

**Methods:**

Qualitative interviews were conducted with primary care physicians in Ontario, Canada who were identified as medium- or high-volume antibiotic prescribers (high volume defined as top 20^th^ percentile versus “medium” defined as 40^th^ to 60^th^ percentile). The interview guide and analysis were informed by the Theoretical Domains Framework. Each interview was coded by two research team members. Sampling and analysis continued until thematic saturation was achieved.

**Results:**

Twenty family physicians were interviewed. Physicians felt that many decisions about prescribing for upper respiratory tract infection symptoms were straightforward (i.e., black and white). However, intention to avoid prescribing in cases where an antibiotic was not indicated clinically did not always align with the provider action or expectation of the patient. Clinical decisions were influenced by the Theoretical Domain Framework domains that were both internal to the physician (*Knowledge, Skills, Social/Professional Role, and Belief about Capabilities*) and external to the physician (*Social Influence, Belief about Consequences, Reinforcement, Emotions, and Behavioural Regulation*). The *Environmental Context and Resources* played a key role. Physicians reported significant differences in their approach to antibiotic prescribing within episodic (walk-in) or continuity of care settings, as the presence (or not) of longitudinal physician–patient relationships seemed to moderate the role of these factors on the decision-making process in cases of uncertainty.

**Conclusions:**

Antibiotic prescribing in primary care is a complex decision-making process in which context may outweigh biology during encounters featuring clinical uncertainty. Differential skill in handling uncertainty and tactics used to operationalize guideline recommendations in the real world seems to contribute to observed variation in prescribing patterns, as much or more than differences in knowledge of best practices.

**Supplementary Information:**

The online version contains supplementary material available at 10.1186/s12875-022-01806-8.

## Background

Deemed a “crisis” almost three decades ago, antimicrobial resistance continues to be a major challenge to modern medicine globally [1], leading to higher levels of mortality and severity of infection, and lost productivity [2]. One driver of antimicrobial resistance is the misuse and overuse of antibiotics in healthcare. The vast majority of antibiotics are prescribed for outpatients [3], with two-thirds of outpatient antibiotics in Canada prescribed by family physicians [3]. One quarter of antibiotics are prescribed for conditions for which they are either rarely or never indicated [4], highlighting that factors beyond clinical presentation are at play [5].

Unnecessary antibiotic prescribing has been associated with practice volume [6], patient-level characteristics (i.e., viral infection, severity of symptoms, age, presence of co-morbidities, race, and previous antibiotic use) [7, 8], and physician-level characteristics (i.e. age, specialty, clinic type, education and years of experience) [9–12]. However, substantial observed inter-physician variability in antibiotic prescribing cannot be explained by differences in patient characteristics [13]. A systematic review of qualitative studies that sought to understand the drivers of physician antibiotic prescribing behaviour described the influence of physicians’ attitudes on prescribing decisions, including complacency and fear, patient signs and symptoms, and time pressure constraints, as most commonly reported factors [14]. While this work is an important first step in understanding the specific determinants of prescribing, it remains unclear when and how these factors operate and whether they are amendable to change.

Interventions to improve appropriateness of antibiotic prescribing have had varying success [15]. Choosing Wisely – a multi-national campaign that aims to reduce unnecessary testing and treatments in healthcare by engaging health care professionals to take leadership in reducing unnecessary tests, try to encourage guideline-concordant prescribing, generally through passive strategies [16, 17]. Reductions in antibiotic prescribing may be achieved by incorporating evidence-based antimicrobial stewardship interventions in the community that are informed by principles of behavioural science, above and beyond disseminating up-to-date clinical guidelines [18]. In a recent study of 3500 high-volume-antibiotic-prescribing family physicians, peer comparison feedback reduced antibiotic prescribing [4]. By better understanding the modifiable determinants contributing to variability in antibiotic prescribing, we are better able to understand which strategies should be deployed, for whom, and in what way. To achieve this, the objective of this study was to identify the determinants of antibiotic prescribing behaviour among family physicians to inform targeted antimicrobial stewardship interventions.

## Methods

### Study design

This qualitative, exploratory, study used semi-structured interviews with a cohort of practicing family physicians in Ontario to elicit their experiences with and perceptions of antibiotic prescribing. This research was conducted in partnership with Public Health Ontario, the agency tasked with providing scientific advice to the provincial government [19]. Research ethics approval was received by the Women’s College Hospital Research Ethics Board [REB #2018–0174-E]. All participants were sent an information letter and informed consent form prior to the interview and were asked to review, sign, and return the form prior to the interview. If written informed consent was not obtained in advance, verbal informed consent was taken before the interview began.

### Setting

In Ontario, family physicians represent the first point of contact for the patient within the health system. In addition to primary care clinics, many family physicians work in walk-in clinics, hospitals, emergency rooms, and/or nursing homes. The notable difference between primary care clinics and walk-in clinics in Ontario, Canada, is the episodic nature of patient visits in walk-in clinics compared to the continuity of care experienced in family practice. Patients may access their own family physician (when they have a family physician) or seek care for acute symptoms from a walk-in clinic if they choose. Family physicians are paid by the government on a spectrum of funding models from capitation-based to fee-for-service [20]. There is no co-pay for patients to see a family physician.

### Sampling and recruitment

A purposeful sampling strategy was used to recruit family physicians that practiced in Ontario and had a high volume of prescribing for both antibiotic and non-antibiotic medications that were eligible to participate. Primary care physicians in Ontario identified as high- or medium-volume prescribers (proxy for practice size) were eligible. Physicians that prescribe the lowest 20^th^ percentile of antibiotics were excluded from sampling as they were likely to provide specialized care or did not practice full time (i.e., low volume prescriber) – our aim was to identify providers that use antibiotics in their practice regularly. This prescribing information was acquired through a dataset that our partners at Public Health Ontario (PHO) accessed through IQVIA, a commercial healthcare company. Eligible physicians were then stratified by (i) volume of antibiotic prescribing, with “high” defined as top 20^th^ percentile versus “medium” defined as 40^th^ to 60^th^ percentile and (ii) years in practice, with “later career” defined as 25 + years since medical school graduation versus “early career” defined as less than 10 years since graduation. Physicians not meeting these criteria were not recruited to allow comparison between groups.

To recruit physicians, a designated analyst at PHO mailed 677 targeted study recruitment letters in total over five rounds between March and December 2019. The analyst was not blinded as they were only involved in recruitment. Additional sampling occurred when participants outside of our criteria came across the recruitment letter addressed to a targeted participant and contacted the study team to complete an interview. As the interviewer was blinded to the participants prescribing status, it did not become known until after the analysis when the researchers were unblinded that two participants did not match the recruitment criteria. These interviews are not used in our comparison but were maintained in the overall analysis.

### Data collection

The team of qualitative researchers, behavioral science experts, and family medicine and infectious disease physicians developed a semi-structured interview guide (Additional 1: Interview Guide) based on the Theoretical Domains Framework (TDF) [21]. The TDF is a validated framework of 84 determinants across 14 domains that is based on psychological theory and used to identify determinants of individual behaviour [21]. The TDF was selected because it offers an overarching theoretical way to understand underlying processes involved in behavior change, which can be mapped to intervention components [21]. Domains include: *Knowledge; Skills; Social/Professional Role and Identity; Beliefs about Capabilities; Optimism; Beliefs about Consequences; Reinforcement; Intentions; Goals; Memory, Attention, and Decision Processes; Environmental Context and Resources; and Social Influences* [21]. The interview guide (Additional 1: Interview Guide) included a combination of questions specific to TDF domains, as well as several clinical case examples designed by physician authors, confirmed by two patient partners, and piloted with a physician not involved in the project. The case examples were designed to explore how physicians decide to initiate, select, and choose the duration of antibiotic therapy for patients presenting with upper respiratory tract infection symptoms. The interview guide was designed to address the research question, using the case examples to work through the physician decision making process, and explore the TDF domains involved in this process.

Prior to recording the interview, demographic questions not available in the IQVIA database were asked including location (urban, rural, remote) and additional practice setting (walk-in clinic, emergency department, long-term care, and other). Telephone interviews were conducted between March-December 2019 by MSi (MSc.), a public health researcher with a background in qualitative methods and no prior relationship with the participants. Interviews were audio recorded and transcribed verbatim by a professional transcription service. Prior to the interview and during initial analysis, the research team remained blinded to whether the participant was a high/medium volume prescriber or early/late career.

### Data analysis

Deductive and inductive analytical processes were conducted initially by MSi, and MSa (MN; Master of Nursing), a nurse-researcher with qualitative methods training. For the former, the TDF was applied as a coding framework to identify determinants of antibiotic prescribing. An inductive process was used to identify codes that did not fit within a TDF domain [22]. Data collection and analysis was an iterative process, which allowed the prompts within the interview guide to evolve along with analysis. Interview transcripts were coded in Excel and themes were developed by the two lead researchers using a codebook and a thematic coding scheme to organize data, with high coder reliability established following the first interviews. We pursued validity in our analyses by having researchers independently code transcripts and having the TDF domains and inductive themes identified by the coders discussed and confirmed with other members of the research team throughout the analysis. Throughout analysis, the research team met repeatedly to refine the coding framework. Thematic saturation occurred between 15 and 17 interviews and an additional 3–5 were conducted to confirm no additional ideas related to the research question were presented, and to meet the pre-established recruitment goal of equal distribution by prescribing level and years of experience.

Member checking to confirm results was conducted in December 2020 by sending a summary of findings to participants who had agreed to further contact by e-mail and asking for their feedback. As the coronavirus disease 2019 (COVID-19) pandemic was thought to be impacting antibiotic prescribing, an additional question was asked (following an REB amendment) in the member checking e-mail to see if participants felt their antibiotic prescribing practices had changed due to the pandemic. No additional data was provided by participants, so no results are presented on this question.

## Results

Twenty family physicians were interviewed. Thirteen participants identified as male (65%), 11 (55%) reported being in practice 10 years or less, and 10 (50%) were high-volume prescribers. Demographic characteristics provided from the IQVIA database or verbally self-reported at the start of the interview are described in Table 1. Interviews lasted between 20 and 80 min (average 30 min).

**Table 1:** Characteristics of family physicians recruited into the study including data from the IQVIA database and self-report

Demographics	Interviews n
# of Participants	20
Gender	
Female	7
Male	13
Years in Practice	
> 10 years since graduation	11
25 + years since graduation	9
Prescribing^a^	
High (top 20^th^ percentile)	10
Medium (40-60^th^ percentile)	8
Low (bottom 39^th^ percentile)	1
Unknown	1
Location	
Urban	19
Rural	1
Primary Care Practice Settings^b^	
^b^Walk-in clinic	12
^b^Long-term Care	2
^b^Emergency Department	2
Continuity primary care clinics	20

^a^ Due to interviewer recruitment blinding, one participant is a low prescriber, and one participant is unknown

^b^ Self-reported; 15 physicians discussed working in more than one practice setting

To unpack the complex antibiotic prescribing decision-making processes in the ‘grey area’ where participants reported uncertainty, TDF domains identified through the analysis were categorized into groupings related to the prescribing decision itself (*Attention/Memory and Decision Making; Intention)*, the factors internal *(Knowledge; Skills; Social and Professional Role; and Belief about Capabilities)* and external *(Social Influence; Belief about Consequences; Reinforcement; Behavioral Regulation; and Emotion)* to the physician that affect that decision. Internal factors were those understood to be playing a role intrinsic to the individual physician making the prescribing decision, while external factors were those that were dependent on others (the patient, other providers, etc.) to inform the decision.

**Fig. 1:**
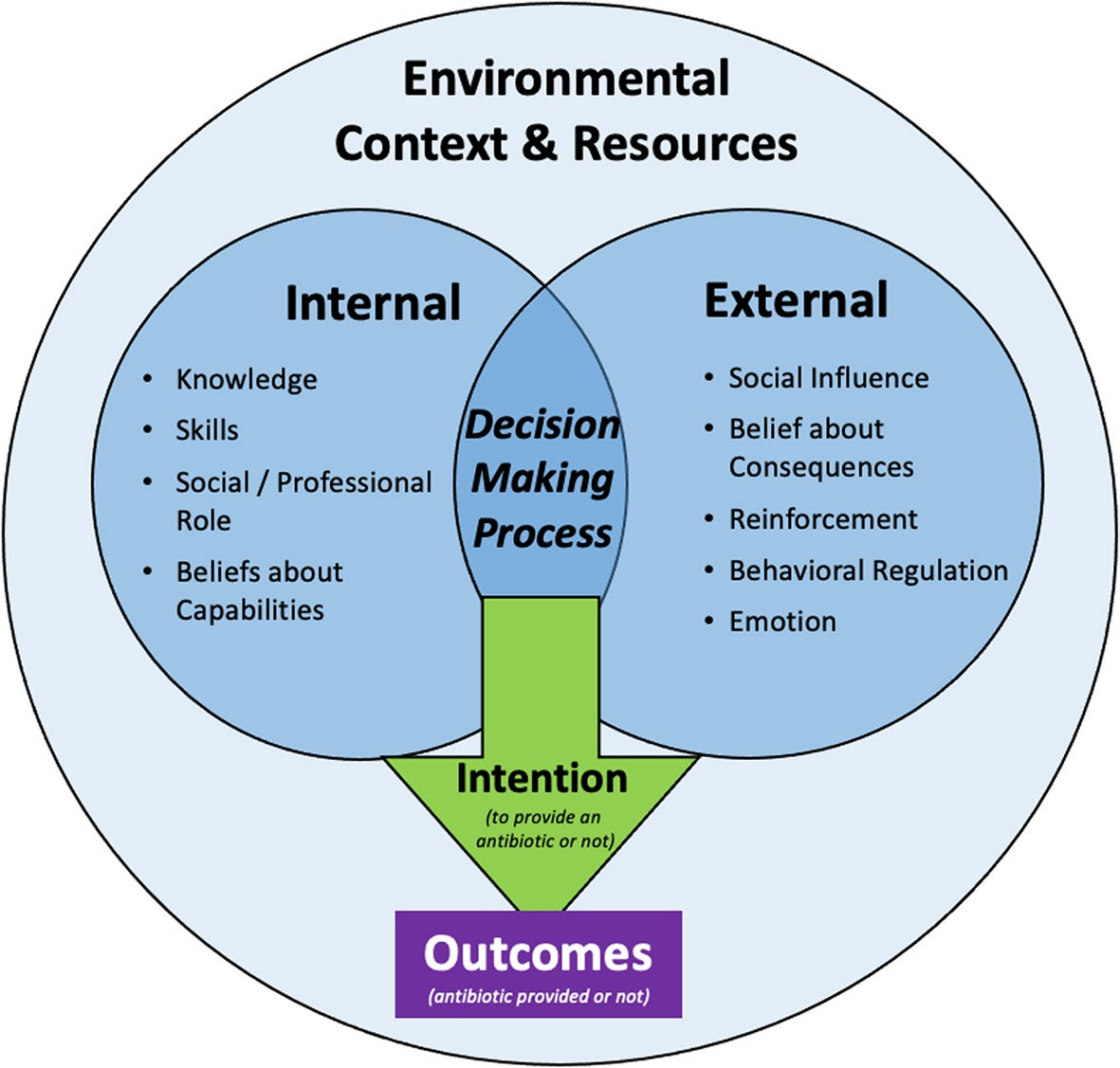
A visual representation of the physicians *Decision Making Process* and the domains that influence the *Intention* to prescribe an antibiotic (or not) to treat respiratory symptoms, leading to the final Outcome. The decision-making process is influenced by internal (*Knowledge, Skills,* etc.) and external (R*einforcement, Social Influence *etc*.*) factors (domains of the Theoretical Domains Framework) that are all impacted by the *Environmental Context and Resources* in which the interaction is taking place

*Attention/Memory and Decision Making* was described as the process the physicians took to achieve an Outcome when they were in the “grey area” of clinical uncertainty on whether to prescribe or not. When the physicians felt clinically uncertain, some physicians reported staying strong with their intended action (i.e., to not prescribe an antibiotic), while others described how various factors, such as pressure from the patient, changed the Outcome. These factors were all understood as occurring within a set of *Environmental Context and Resources*, such as whether the interaction took place in an episodic care setting (i.e., a walk-in clinic) or a traditional family practice. A visual representation of the decision-making process is provided in Fig. 1.

### The role of clinical context

All physicians reported that when they felt clinically uncertain (in the ‘grey area,’), *Environment Context and Resource* had an overarching impact, especially the clinical context in which care was provided (i.e., walk-in clinic versus family practice with continuity of care). This context was reported by participants to moderate their antibiotic prescribing behavior based on access to diagnostic testing, clinical characteristics of patients, time pressure, and ability to develop patient rapport.

*“But it is hard sometimes in the walk-in, the volume, you’re going, you’re tired, and people are cranky, and they’ve been waiting for three hours. But I really try to not give antibiotics.”* P8 [Medium Prescriber; Early Career].

Many physicians that practiced in episodic care settings as well as traditional family practice settings discussed the impact of the patient-provider relationship and patient volume on their final decision. The existence of a strong patient-provider relationship was felt to reduce patient expectations for antibiotics (real or perceived) by giving more weight to the provider’s expertise, thus allowing the provider to be more resolute in their decision to withhold an antibiotic when it was not indicated.

*“I’ve been in practice so long now so my patients kind of know my style. … But I always say, if you get worse in any way, if you develop a fever after today, I want you to call me and come back and be reassessed to see if I change my treatment plan.”* P13 [Medium Prescriber; Later Career].

Many physicians described the presence of patient “trust” in their prescribing practice, which they felt was strengthened by either years in practice or self-perceived expertise, and mainly available in settings with continuity of care. *“It’s easier when it’s your patient because you already have some trust built up with them.”* P4 [High Prescriber; Early Career] In episodic settings or situations with no prior relationship and increased time pressure, many physicians reported feeling less confident in their ability to avoid antibiotics. One high prescriber acknowledged this difference by comparing their prescribing practices in their own clinic to when they worked at the walk-in clinic.

*“In my own practice, there’s a level of trust, and there’s also accessibility. So, if I instruct the patient with, I don’t think you need an antibiotic right now but I’m here all week, you know how to get a hold of me, you can always come back… I do it all wrong there* [at the walk-in clinic]*… I readily admit that I inappropriately prescribe…sometimes if it’s super busy, I’m run off my feet, I can tell this person is hell bent on an antibiotic for their cough of three days duration, no fever, I’ll do it.”* P20; [High Prescriber; Later Career].

Patient characteristics (age, cultural background, co-morbidity, type, and severity of symptoms) were also reported to vary between clinic settings, with patients presenting to episodic care settings with more acute needs. In this context, physicians felt that patient pressure and expectations were more influential, with all of these factors coming together to create the “perfect storm” which increased antibiotic prescribing.

“*The problem with a walk-in, you don’t establish a relationship with the patient that you get to see once. They see you as a walk-in doctor. ‘I’m only going to see you once, I don’t really care what you say.’ You know, that kind of thing. ‘I’m here for that, this is what I want, this is what my doctor would have given me’, you know. And I think that’s where the major difference is.”* P15; [Medium Prescriber; Later Career].

### Internal factors: beliefs that drive them and the skills needed to manage them

Physicians mentioned the internal factors impacting their decision-making process in the ‘grey area’ included *Knowledge, Skills, Social/Professional Role, and Belief about Capabilities.* Physicians were typically confident in their knowledge and abilities *(Knowledge; Skills; Belief about Capabilities)* to diagnose and prescribe appropriately for cases presenting with upper respiratory symptoms, although some acknowledged *Knowledge* gaps or recent learning regarding optimal duration of antibiotic prescribing. *“I’ve been following more recent evidence that suggests that less* [antibiotics] *is more. So, a 5 to 7-day course instead of 7 to 14.”* P11 [High Prescriber; Early Career] Physicians did not have a consistent strategy or threshold for weighing the risk of harms from unnecessary antibiotics.

In cases of diagnostic uncertainty, some physicians reported that they might prescribe an antibiotic with the hope of both managing symptoms and preventing the condition from worsening, which was perceived to outweigh the risk of antibiotics. Others were more adamant in only prescribing when indicated. “*Bronchitis is a viral infection. I do not prescribe antibiotics for that. It’s very extremely rarely that it could be bacterial, so I tell the patient if they feel that they’re feeling worse to come back in and get checked again, and then at that point, we might want to consider antibiotics.”* P2 [High Prescriber; Later Career].

When in the grey area, the physicians tended to justify the decision to prescribe an antibiotic in various ways, such as, ‘the patient is old and frail’, the ‘patient has a known complex history’, ‘patient cannot access care easily due to mobility’. Many physicians felt tension between their perceived *Professional Role* to prioritize doing what was right for the patient while also mitigating the population-level risks of antimicrobial resistance.

*“I think at the end of the day, you have to do the best for the patient, and that will do the best for the population. Sometimes, doing the best for the patient is not giving them antibiotics unless they need it, and that will ultimately help with antibiotic resistance.”* P7 [Medium Prescriber; Early Career].

Physicians knew they needed to limit antibiotic use *(Knowledge)* but had to have the *Skills* to balance this with doing what they perceived was needed for the patient sitting in front of them. Some physicians felt that in certain situations it was their *Professional Role* to protect healthcare dollars by prescribing the antibiotic, as they perceived the patient would go elsewhere for the prescription, as described by one high prescriber.

*“I would tell them I don’t think they need to take it, and I’m only giving it to them because they’re saying they’re going to go to another doctor, and they’re going to keep going to another doctor until they get antibiotics. So, it’s a waste of healthcare dollars for them to keep bouncing from doctor to doctor, right?”* P2 [High Prescriber; Later Career].

Sometimes, when physicians felt the indication to prescribe seemed clear (“black and white”) based on clinical symptoms alone, the outcome of the encounter did not always align with the physician’s initial *Intention* when faced with other complicating factors. Physicians described using their inter-personal and communication *Skills* to educate the patient and to convert their *Intention* to not prescribe into an aligned outcome, by building trust with patients or effectively explaining the decision. Some physicians (sometimes associated with years in practice) described using a routinized approach to explain to their patient that antibiotics were not indicated (i.e., their “spiel”).

*“Just saying* [to the patient], *‘antibiotic resistance is rising. We’re really trying to cut back on our prescribing. There’s always risk to antibiotics, so it could give you diarrhea, C. diff, put you in the hospital. There’s lots of consequences to giving antibiotics when they aren’t warranted.’ That’s the spiel I give…You’ve got to feel out which person you have to give that … a little bit more information to because they’re not as receptive or understand the reasons behind not giving antibiotics to everybody.”* P8 [Medium Prescriber; Early Career].

However, particularly in episodic settings, some physicians did not feel their explanation *(Skills)* were enough and they lacked *Belief about their Capabilities.*

“*There were so many times* [in a walk-in] *where it was like, you know what, why am I going through all this spiel. Because, at the end of it, after spending 15 min with the parents or with the patient, they’re just going to say, so what antibiotics are you going to give me.”* P15 [Medium Prescriber; Later Career].

When the physician lacked confidence in their ability to convince patients that antibiotics were not warranted (*Beliefs about Capabilities*), many described using a “wait-and-see” approach, such as delayed prescribing, sometimes providing a prescription even against their better judgment.

*“If parents are fairly insistent that they need antibiotics, that’s when I’ll try and use the antibiotic prescription to-go. So, more of a wait and see approach. Even though I’m fairly certain it’s likely a virus and it will resolve on its own with more time”* P9 [Medium Prescriber; Early Career].

Physicians with more experience (self-reported, not necessarily later career), those that perceived themselves to have strong clinical judgement, and those with long-standing relationships with patients in their practice, explained how the resulting confidence (an increase in *Belief about Capabilities*) made it easier to provide guideline-concordant antibiotic prescribing.

*“I mean, maybe, five years ago, I would have been like, okay, fine, and now I’m like, no, I’m not going to give you antibiotics if it doesn’t make sense. I feel more confident to be firm in my convictions and my assessment of patients.”* P7 [Medium Prescriber; Early Career].

### External factors influencing the decision-making process

External factors impacting the physician decision-making process included: *Belief about Consequences, Emotions, Social Influence, Reinforcement, and Behavioural Regulation*. During times of clinical uncertainty, some physicians emphasized the importance of antibiotics to prevent worse outcomes when the patient presentation was complicated by medical (i.e., multiple co-morbidities) or social (i.e., recent immigrant or isolated older adult) factors (*Beliefs about Consequences*).

“…*there are a lot of smokers, COPD, asthma. For those patients that do have symptoms, they usually require an antibiotic and steroid. Those patients I literally treat with antibiotics. Whereas another patient who doesn’t have any lung disease or any other issues, then I try to avoid them *[antibiotics]* at all costs if possible*” P5 [Medium Prescriber; Later Career].

Some physicians discussed encounters they felt were more challenging to navigate, such as when the patient was perceived as becoming angry, demanding, or threatening (*Emotion*), leading to some cases where physicians felt they had to “give in” to meet the perceived patient expectations (*Social Influence*).

*“If a patient in the walk-in clinic says … their opening words are, I have bronchitis, I need an antibiotic, well, you’ve pretty much lost the battle already. So, either, even if you educate this patient, they’re probably going to walk away disgruntled, unhappy*.” P20 [High Prescriber; Later Career].

*Social Influence* was also discussed by some physicians who felt a lack of support from their peers regarding antibiotic prescribing. For example, some physicians commented that it was uncommon to discuss antibiotic prescribing decisions with anyone. One early career physician described being uncomfortable talking to a later career physician who they felt was likely a high prescriber.

*“I don’t feel comfortable going to talk to somebody 25 years older than me and tell them what they’re doing is wrong or what I think is wrong for example.”* P4 [High Prescriber; Early Career].

*Reinforcement* was discussed as an external factor in connection to *Environmental Context and Resources,* as when physician’s reported access to resources such as diagnostic testing, X-rays, or having an Electronic Medical Record (EMR) system with patient history, including easily accessible history of antibiotic prescribing, this access was reported to influence and *Reinforce* their clinical decision and their confidence in that decision. Physicians mentioned that these tests could also satisfy the patient to some extent in situations of pressure or demand for an antibiotic even if an antibiotic is not provided.

*“I feel like I need a clear indication. For strep, I have a positive strep because we have the test for that and pneumonia, I have a positive X-ray. … I like a clear indication for why I’m giving the antibiotics because of the side effects and the resistance.”* P8 [Medium Prescriber; Early Career].

*Reinforcement* was also identified as many physicians mentioned using patient-facing resources, such as Choosing Wisely pamphlets [17], and symptomatic treatment as an alternative to an antibiotic, as these tools helped reinforce the physicians decision, and the patient would leave feeling that their symptoms were being taken seriously. Physicians repeatedly mentioned the Choosing Wisely campaign [17] as a supportive resource that helped them achieve guideline-concordant antibiotic prescribing *(Reinforcement).* This type of resource was trusted by many physicians and was also thought to act as reinforcement to the patient that the provider’s instruction was in line with the accepted standard of care.

*“My understanding is that Choosing Wisely Canada now is accepted as a standard of care. So, I think that is very reassuring and gives me, at least, a lot more confidence in my clinical practice… They* [Choosing Wisely] *have these nice posters, so a lot of patients sitting, when they glance at posters on the wall in front of them, they have that sense before I walk in the room that maybe this is not one of the clinics that I'm going to get antibiotics.”* P3 [High Prescriber; Early Career].

A final topic discussed by physicians was *Behavioral Regulation*, described as strategies used by physicians to monitor their own prescribing practices. Most physicians reported being open to receiving feedback about their prescribing, and several had already signed up to receive reports about their prescribing practices [23] or had received an individualized letter (part of a separate study) [4]. Based on this feedback, some physicians reported no change, while others reported increased awareness or even that they had changed their prescribing practice based on this feedback.

“*I remember I was surprised, disappointed, and it definitely, kind of, firmed up my resolve to be a little bit more restrictive in my prescribing* [when received feedback about their antibiotics prescribing]. *I can tell you that the information I did receive changed my practice to some extent. So I did not feel like that was inappropriate. I mean, being compared to your peers is always motivating. Because when you compare it to a study, you can always say, well, my patient doesn’t meet this study criteria. But I can’t convince myself that, oh, I’m seeing sicker people than my neighbouring physician…… it’s* [the feedback] *just made me a little more resolute in saying, no, this is viral, no antibiotic.* P11 [High Prescriber; Early Career].

## Discussion

This study highlights that increasing *Knowledge* may be a less-important target for antimicrobial stewardship than interventions that help clinicians apply that knowledge during times of clinical uncertainty and when facing various pressures or constraints. The decision-making process for prescribing antibiotics in primary care involves each physician balancing multiple, complex, interacting, internal and external factors that affect their *Beliefs about Consequences* and *Beliefs about Capabilities*, and that require advanced communication and interpersonal *Skills* to navigate. The *Environmental Context and Resources*, particularly a clinical setting emphasizing either episodic care or continuity of care with longitudinal patient relationships, interact with these factors along with available resources (diagnostic testing, support staff, Choosing Wisely tools, etc.), to enable (or interrupt) delivery of guideline-concordant antibiotic prescribing.

To navigate these complex factors during decision-making in the “grey area”, physicians seem to develop heuristics to enable expedient decisions about when to deviate or not from perceived guideline recommendations during real-world encounters with patients seeking treatment for acute respiratory symptoms. These practical problem-solving strategies (i.e., “Mindlines”) [24] may eventually become a standard and unconscious element of practice—a habit. Other studies have demonstrated that a range of factors related to the patient and their situation have an impact on clinical decision-making [25–27]. In line with these other studies, physicians explained prescribing as a conscious weighing of risks, judging that, on the whole, prescribing antibiotics represented the lowest risk option [14]. In other instances, antibiotics were prescribed due to a belief that it would not be possible to effectively argue for alternative treatment in a short interaction with a patient. In our study, heuristics about when to deviate from guidelines by prescribing antibiotics seemed to be associated with years in practice and practice volume, suggesting that the conscious weighing of alternatives can be superseded by habits. Prior studies agree on the important role of time pressure in that it creates circumstances where heuristics are more likely to be unconscious to avoid difficult interactions [14]. These factors can hold more or less weight on the *Decision-Making Process *depending on the clinical context in which the provider is practicing [27, 28]. In the UK, similar research exploring high, medium, and low prescribers reinforces the patterns found in this study [29]. Medium prescribers in our study tended to emphasize shared decision-making experience and skills often gained through years in practice or exposure to more acute respiratory cases due to clinical context [29].

Unfortunately, there is currently little consideration of the broad range of behavioural determinants when designing interventions to reduce antibiotic prescribing [30, 31]. Feedback has a role to play [4] to help prescribers understand that their performance may not be in line with their goals, but provision of prescribing data alone will not address the multiple and complex determinants identified in this work. The findings highlight the importance of strong interpersonal and communication *Skills* to help physicians navigate patient encounters in which their clinical decision (*Intention*) to prescribe an antibiotic (or not) does not align with the perceived patient preference. Thus, antimicrobial stewardship efforts in the community should feature communications training and skills development to support physicians in navigating and negotiating with patients, rather than just reviewing the guideline recommendations for initiation and indication. Additionally, guidelines might be more helpful if they integrate advice for managing scenarios which commonly lead to diagnostic uncertainty and if they were accompanied by evidence-based implementation aids such as recommendations for when and how to implement delayed prescriptions [29, 32–34]. This study suggests that the duration of antibiotics prescribed in outpatient clinic settings is most amenable to improvement, particularly for providers that had been in practice for longer [35–38]. Results also suggest that the decision to prescribe is based on a rapidly accessed heuristic but the decision about what to prescribe and for what duration is more purposeful. If this is true, point-of-care decision support tools may be useful to cue prescriptions that are shorter and for narrower spectrum antibiotics, but strategies must focus on the determinants described here-in to change established heuristics on whether to prescribe.

Some physicians feel ill-equipped to engage with patients about the appropriate use of antibiotics [39, 40]. Interventions must support physicians in addressing both real and perceived needs and expectations for antibiotics, especially given that physicians tend to over-estimate patient desire for antibiotics [41, 42]. Such strategies may be more beneficial for providers if they include inter-personal and shared decision making strategies to support providers to engage in challenging conversations and build confidence in educating their patients to avoid an inappropriate antibiotic [29]. Other key domains (including *Environmental Context and Resources*, *Social Influence*, and *Skills*) highlight important targets for future interventions seeking to change antibiotic prescribing [25, 28]. Such interventions could be tailored for contexts without trusting clinical relationships and with high patient volumes. System-level interventions that enable continuity and discourage use of ‘walk-in’ type care may help with inappropriate prescribing, especially if coupled with public health communication about antibiotic harms. An example of the latter is the Choosing Wisely campaign ‘using antibiotics wisely’ that targets both providers and patients with the same consistent messaging [17]. Interventions could ideally be designed not only to teach physicians how to best use antibiotics but also how to educate their patients and the public to break the cycle of expectation and demand [43–47].

### Limitations

The sample was limited to family physicians in Ontario. While we successfully recruited later and earlier-career physicians as well as high and medium volume prescribers, we were only able to recruit one rural family physician. As is the case in most studies of this nature, selection bias is also possible, since only those who were willing and interested responded to our request and those who did not engage may have different perspectives. As interviews were conducted prior to the COVID-19 pandemic, practice patterns may have changed, especially with the increasing use of virtual care rather than in-person consultations. To account for this change, as part of our member checking process, we contacted participants to explore whether their approach to antibiotic prescribing had changed but no response was provided to include for consideration and analysis.

## Conclusion

When physicians encounter tension between the idealized, guideline-concordant clinical decision and the perceived needs of the specific patient in their clinic, they either rationalize a change to their own clinical decision or recruit substantial cognitive resources to try to convince the patient of the guideline-concordant approach. Multiple domains play a role in this *Decision-Making Process* in this “grey area”, particularly *Environmental Context and Resources*, where physicians reported significant differences in their antibiotic prescribing depending on if it were an episodic (walk-in) or continuity of care (family practice) model. Strategies to reduce inappropriate antibiotic prescribing need to move beyond increasing *Knowledge* to include strategies for managing uncertainty, and addressing how to apply guidelines in situations with uncertain diagnoses or patient pressures.

## Supplementary Information

**Additional file 1: **Interview Guide.

## Data Availability

The datasets generated and analyzed during the current study are not publicly available as it is qualitative data that can be identifiable but may be available from the corresponding author on reasonable request.

## Abbreviations

PHO: Public Health Ontario
EMR: Electronic Medical Records
TDF: Theoretical Domains Framework
