# Supplementary material for: Coping with ‘the grey area’ of antibiotic prescribing: a theory-informed qualitative study exploring family physician perspectives on antibiotic prescribing
Source: BMC Prim Care. 2022 Jul 28;23:188. doi: 10.1186/s12875-022-01806-8 (PMC9330951; doi:10.1186/s12875-022-01806-8)
Supplement: Supplementary file 1 — Additional file 1: Interview Guide. [file 12875_2022_1806_MOESM1_ESM.docx]

**Appendix 1: Interview Guide**

**Interview Questions**

**Part A: Demographic Questions**

1. What is the average number of patients you would see in a full day?
2. How many days do you have outpatient clinics per week?
3. How many “same day” slots do you have in a typical week? (Prompt: open slots for emergency/urgent visits)
4. How many urgent-care or walk-in type clinics do you have in a typical week?
5. How many doctors work in your practice? How many pharmacists?
6. Are you affiliated with a university? Yes/No

**Part B: Antibiotic prescribing in practice**

***I am going to outline 4 similar but distinct scenarios here related to antibiotic prescribing in your practice, okay? We will work through these scenarios one by one but please stop me at any point if you have questions or need clarification.***

**Scenario 1:**

1. Can you talk me through a time when a patient over the age of 70 presented with upper respiratory tract infection symptoms and you prescribed an antibiotic? Can you talk me through the patient encounter and your thought processes in that particular case?
   1. What factors influenced you to provide an antibiotic?

Prompt: time of day, day of the week?

- 1. What factors influenced how long the antibiotic was prescribed for?
  2. Would you make the same decisions again? Why?
  3. If the patient was a child, how does that change the clinical scenario?
  4. If the patient was an adult, how does that change the clinical scenario?

IF PARTICIPANT SAY’S THIS HAS NEVER HAPPENED: Imagine the patient was diagnosed with bronchitis. Can you talk me through how your clinical thinking might change? What if the patient was a child?

**Scenario 2:**

1. Consider if a patient over the age of 70 presented with upper respiratory tract infection symptoms and you did not prescribe an antibiotic? Can you talk me through the patient encounter and your thought process in that particular case?
   1. What factors influenced you not to prescribe an antibiotic?

Prompt: age, timing, shared decision making?

- 1. Would you make the same decision again? Why?
  2. Did you explain your thought process to the patient? If so, how?
  3. If the patient was a child, how might that change the clinical scenario?
     1. Follow-up: What if it was an adult man/woman?
  4. Imagine the patient was diagnosed with bronchitis. Can you walk me through how your clinical thought process might change?

**Scenario 3:**

1. Can you talk me through a time when you did not want to prescribe an antibiotic to a patient with upper respiratory tract infection symptoms but ended up prescribing it anyway?
   1. Can you describe the conversation you had with the patient?
   2. How did the patient react?
      1. Did the patient’s reaction influence your thought process? If so, how?
   3. What factors influenced you to provide the antibiotic?

Prompt: contextual or situational pressure?

- 1. Would you make the same decisions again? Why?

Prompt: Change initiation? Change duration?

- 1. Have you had this experience when the patient was a child or an older adult? If so, what was different about this process?

**Scenario 4:**

1. In situations where the need for an antibiotic prescription was not clear, would you ever discuss whether an antibiotic is required with anyone on your clinic team?
   1. If so, can you talk me through that discussion?

Prompt: Did it inform your practice?

- 1. What were the benefits of engaging in this discussion?

Prompt: Personal? Clinic level? Facilitators during the conversation?

- 1. What were the challenges of having this discussion?
  2. Have you ever discussed *your general approach* to antibiotic prescribing with others, such as those in your clinical team?
     1. If so, can you talk me through that discussion?
     2. What were the challenges of the discussion?
     3. What would have facilitated the discussion?
  3. If NEVER speak with anyone about a specific case or general antibiotic prescribing:
     1. Why not?
     2. What would encourage you to discuss antibiotic prescribing with others?

1. Would you be interested in receiving individualized data about your antibiotic prescribing practices?

If YES:

- 1. Why? What would you do with that information?
  2. What data would you want to see?

Prompt: Initiation? Duration? Frequency? Selection?

- 1. Would you want to see how your data compares to your peers? How would you like to receive that information?

Prompt: A report, such as those from Health Quality Ontario

- 1. Do you think you would be comfortable discussing that information with your peers? Why?

**Part C: Individual and population impact of prescribing antibiotics**

1. To what extent is your thought process to prescribe or not prescribe an antibiotic influenced by the potential side effects of antibiotics? Why? By antimicrobial resistance at the population level? Why?
   1. Are you aware that by 2050 there will be 10 million annual deaths due to antimicrobial resistant infections, more than diabetes and cancer combined? Does or would information such as this impact your decision(s)? Why?

(Source: UK Prime Minster commissioned Review on Antimicrobial Resistance, supported by the Wellcome Trust and the UK government).

- 1. Considering this, do you weigh the benefits of prescribing an antibiotic to a patient against the potential negative impact at the patient level? Population level? Or both? How might you do this?
  2. Does your general approach differ for older adults? Children? Other specific populations?
  3. How do you define/determine/distinguish URI symptoms versus a diagnosis? Does your thought process to prescribe differ based on a clinical diagnosis? (bronchitis, sinusitis etc.)
  4. Does your thought process change depending on demographic characteristics of the patient?

1. To what extent do you feel you have a role to play in helping to reduce the use of antibiotics in your clinic? Why?
   1. What about more globally? Why?
      1. *If they say NO role: flip the question. To what extent could you contribute to AMS in your clinic or community?*
2. Have you come across any good (or bad) strategies to address the inappropriate prescribing of antibiotics?
   1. Can you think of any ways this could be improved or expanded to meet individual *and* population needs?
   2. What techniques do you use/have you used previously in an attempt to avoid prescribing of antibiotic to patients presenting with upper respiratory tract infection symptoms, when necessary? For older patients? Children?

If NO:

- 1. Why not?
  2. What would encourage you to receive this data?

1. Is there anything else about your unique clinical practice or patient population that we should know about? Is there anything else you would like to add that you think has not been covered by my questions?

Thank you again for taking the time to speak with me.
